# Blood Source and Anesthetics Effects on the Maintenance of *Anopheles darlingi* in the Lab-Rearing Condition

**DOI:** 10.3390/insects16030281

**Published:** 2025-03-08

**Authors:** José Daniel Costa Pontual, Natália Vitória Coelho, Najara Akira Costa dos Santos, Alessandra da Silva Bastos, Jéssica Evangelista Araújo, Alice Oliveira Andrade, Jansen Fernandes Medeiros, Maisa da Silva Araujo

**Affiliations:** 1 Plataforma de Produção e Infecção de Vetores da Malária (PIVEM), Laboratório de Entomologia, Fiocruz Rondônia, Porto Velho 76812-245, RO, Brazil; jose.pontual@fiocruz.br (J.D.C.P.); najara.akira@fiocruz.br (N.A.C.d.S.); alessandra.bastos@fiocruz.br (A.d.S.B.); jessica.evangelista@fiocruz.br (J.E.A.); aliceandrade@usp.br (A.O.A.); jansen.medeiro@fiocruz.br (J.F.M.); 2 Instituto Nacional de Epidemiologia da Amazônia Ocidental (INCT-EpiAMO), Porto Velho 76812-245, RO, Brazil; 3 Programa de Pós-Graduação em Biologia Experimental, Fundação Universidade Federal de Rondônia, Fiocruz Rondônia, Porto Velho 76812-245, RO, Brazil; nataliavitoriac26@gmail.com; 4 Programa de Pós-Graduação em Saúde Pública, Faculdade de Saúde Pública, Universidade Federal de São Paulo, São Paulo 01246-904, SP, Brazil; 5 Laboratório de Pesquisa Translacional e Clínica, Centro de Pesquisa em Medicina Tropical (CEPEM), Porto Velho 76812-329, RO, Brazil

**Keywords:** blood feeding, colony, colonization, *Anopheles darlingi*, malaria vector

## Abstract

*Anopheles darlingi* mosquitoes are the main malaria vectors in the Amazon region and play a significant role in the high transmission cycle of *Plasmodium vivax*, particularly in areas undergoing degradation of the Amazon. Establishing an *An. darlingi* colony under laboratory conditions allows for critical studies on this vector, including insecticide resistance, vector competence, and the development of new tools for controlling vivax malaria. However, the establishment of mass-rearing mosquito colonies has proven challenging, with success being heavily dependent on supporting their reproduction. A key factor in this process is finding the most efficient blood source for feeding. Here, we evaluated the reproductive potential of *An. darlingi* using different blood feeding methods and sources. First, we assessed the effect of anesthesia on reproductive potential by comparing anesthetized mosquitoes with those that were physically restrained. Next, we assessed the best blood source using both direct and indirect blood feeding methods, the latter involving an artificial feeding system. The blood sources tested included from rabbits, chickens, mice, bovines, and humans. In the anesthesia tests, no significant differences in the evaluated biological parameters were observed between anesthetized or non-anesthetized groups. Similarly, no significant differences were detected in the biological parameters assessed for each blood source, regardless of whether the feeding was direct or indirect. Because all blood sources proved effective, the practicality of obtaining and maintaining blood becomes a crucial factor. In this regard, bovine blood emerged as an effective and practical alternative for maintaining an *An. darlingi* colony under laboratory conditions.

## Simple Summary

Female mosquitoes require blood for their egg development and the production of subsequent generations. Establishing a successful mosquito colony in the laboratory involves finding the most effective blood meal source to support reproduction, which can be challenging. In this study, we compared different blood sources and feeding methods. Our findings demonstrate that bovine blood serves as an effective alternative for maintaining *An. darlingi* colony under laboratory conditions, eliminating the need for direct blood feeding on live animals and their maintenance within the institution.

## 1. Introduction

Mosquitoes of the genus *Anopheles* are the vectors responsible for transmitting *Plasmodium* species that cause human malaria. Human malaria can be caused by at least seven *Plasmodium* species (*Plasmodium falciparum*, *Plasmodium vivax*, *Plasmodium malariae*, *Plasmodium ovale wallikeri*, *Plasmodium ovale curtesi*, *Plasmodium knowlesi*) [1]. Malaria remains a significant burden on global public health, with 263 million cases worldwide in 2023 [2]. In the Americas, 548,000 malaria cases were reported in the same year, 72.1% of which were caused by *P. vivax*. Brazil, the Bolivarian Republic of Venezuela, and Colombia accounted for 76.8% of these cases, totaling over 420,000 cases in 2023 [2].

The high incidence of vivax malaria in Brazil and other South American countries poses a major public health challenge. The unique life cycle of *P. vivax*, including the formation of hypnozoites—which can lead to relapses and multiple episodes—and the early production of gametocytes facilitate sustained transmission [3]. The burden of malaria episodes can also result in cognitive impairment and behavioral changes, even in non-severe cases, affecting child development and school performance in endemic areas [4,5,6]. Within the One Health framework, vivax malaria negatively impacts the sociocultural development of populations in endemic regions [5] and imposes a substantial economy burden at the household level due to lost productivity during illness [7].

Despite the significant impact of vivax malaria in endemic regions, the absence of a continuous culture of *P. vivax* parasite in the laboratory limits advances in the research and development of new strategies and tools to control its transmission [8]. As a result, studies on vivax malaria are often restricted to endemic regions. Establishing colonies of anopheline mosquitoes involved in the natural transmission of *P. vivax* is essential for advancing research on *Plasmodium* transmission and the dynamics of vivax malaria [9,10,11,12].

The *An. darlingi* species is one of the main malaria vectors in the Amazon region, especially in areas undergoing degradation, where it plays a key role in the transmission of *Plasmodium* [13,14,15]. Establishing colonies of this vector species under laboratory conditions has enabled the study of various aspects of its biology, including behavior [16,17], physiology, and rearing protocols [18], as well as insecticide resistance [19], vaccines, and antimalarial compounds trials [20,21,22]. It also may facilitate research into taxonomy [23], susceptibility to pathogens [24], and parasite–host interactions [24,25]. Additionally, it enables the evaluation of potential control tools, such as testing new insecticides, monitoring resistance, and developing alternative methods [26,27].

Despite the importance of maintaining mosquito colonies, rearing anopheline mosquitoes under laboratory conditions can be challenging, mainly due to difficulties related to species that do not exhibit stenogamous mating behavior that requires restricted spaces [28,29,30]. Additionally, establishing a successful mosquito colony involves finding the most effective blood meal source and feeding method to support successful reproduction [31,32].

In the laboratory setting, mosquito colonies are often maintained by direct blood feeding from live animals, such as mice, rabbits, guinea pigs, hamsters, or birds [32,33,34]. While live animals provide a constant and appropriate blood source for egg production, direct blood feeding has several drawbacks. These include the high costs associated with maintaining bioteriums, the need for trained technicians to handle the animals, and ethical considerations regarding animal use under laboratory conditions [35,36]. In terms of animal maintenance, another disadvantage of direct blood feeding is the handling of live animals. Even when managed by professionals, these animals may exhibit irritable behavior and become increasingly unstable. The use of anesthesia facilitates easier handling and reduces the risk of pain, discomfort, stress, and exhaustion for the animal [37]. On the other hand, an indirect blood-feeding method, or artificial feeding, mimics natural blood-feeding processes by using the synthetic membrane attached to glass feeders or electronic delivery systems. This approach allows for the use of blood from slaughterhouses, such as birds or bovine blood, eliminating the need to maintain live animals in a bioterium [36,38,39].

In 2018, a lab-reared colony of *An. darlingi* was established at the Malaria Vectors Production and Infection Platform, known as PIVEM, at Fiocruz Rondônia [11], in collaboration with the group that first established a colony of *An. darlingi* in Peru [9], to support malaria research in the Brazilian Amazon. Initially, blood feeding for *An. darlingi* was carried out using direct blood feeding on chickens [11], followed by rabbits. However, the drawbacks of using live animals for blood feeding led to research into alternative blood sources and feeding methods for maintaining an *An. darlingi* colony.

In this study, experiments were conducted to evaluate the effects of blood source and anesthetics on the reproductive potential of *An. darlingi*, as the reproductive potential of females is a critical factor for colony maintenance. Reproductive potential was assessed using the following parameters: (i) blood-feeding rate, (ii) post-blood-feeding female survival rate, (iii) fecundity, (iv) fertility, and (v) larval development through to adult emergence. The study hypothesized that the blood source could influence these reproductive parameters and that anesthetics might also impact them.

## 2. Materials and Methods

### 2.1. Study Design

The *An. darlingi* colony was initially maintained through weekly direct blood feeding on rabbits and chickens, which were physically restrained during feeding sessions. Anesthetics can be used to minimize or prevent pain and stress in animals during laboratory experimentation and direct blood feeding of insect vectors. Therefore, in the first phase of this study (i), the effect of anesthesia on the reproductive potential of *An. darlingi* was evaluated using rabbits and chickens. In this experiment, the animals (rabbits and chickens) were either anesthetized or physically restrained to allow for mosquito feeding.

In the second phase (ii), the most effective blood source was evaluated under two experimental conditions: (i) direct blood feeding on animals and (ii) indirect blood feeding using a membrane feeding system. Due to logistical constraints, animal characteristics and behavior, as well as availability, the direct blood feeding experiment compared blood sources from rabbits, chickens, and humans. For indirect blood feeding tests, the following blood sources were used: rabbit, chicken, mouse, bovine, and human. All blood-feeding procedures were conducted in duplicates and repeated three to five times on different days, with the exception of the indirect blood feeding experiments, which lacked replication. All raw data are available in the Supplementary Materials.

### 2.2. Blood Source

The blood sources used in this study were selected based on *An. darlingi* preferences and their availability in the bioterium of Fiocruz Rondônia. Previous studies have shown that the *An. darlingi* population in Peru display avian host-feeding patterns [40], while the *An. darlingi* population in the Brazilian Amazon commonly feed on non-human mammalian hosts, such as cattle, pigs, and dogs [41,42,43]. Mouse and rabbit blood are widely used to maintain colonies of other mosquito species [36], while human blood was used in this study because it is the preferred source for *An. darlingi* in natural conditions [40,42].

The mice (*Mus musculus*) and rabbits (*Oryctolagus cuniculus*) used in this study were obtained from the Institute of Science and Technology in Biomodels at Fiocruz (ICTB—Fiocruz campus in Manguinhos, Rio de Janeiro, Brazil). Chickens (*Gallus gallus domesticus*) were purchased from a local farm (Granja Aviron, Porto Velho, Rondônia, Brazil). All animals were maintained in the Fiocruz Rondônia Bioterium, and procedures followed the guidelines of the Brazilian College for Animal Experimentation (COBEA). Animal use was approved by the Committee of Ethics in Animals Use (CEUA) at Fiocruz Rondônia, under protocol 2019/10 (November/2019 to December/2022).

Bovine blood (*Bos taurus*) was obtained from a local meatpacker (Areia Branca meatpacker, Porto Velho, Rondônia, Brazil), which operates under state inspection services (Agrosilvopastoral Health Defense Agency of the State of Rondônia—IDARON) and complies with municipal, state, and federal regulations (Ministry of Agriculture, Livestock and Supply—MAPA), including environmental laws. The meatpacker holds a quality certificate from the municipality. The bottle used to collect the blood was previously autoclaved, and 60 µL of anticoagulant (HEPAMAX-S 5000 U.I/mL, 22021550, Blu Farmacêutica S. A., Cotia, São Paulo, Brazil) was added to every 100 mL of the bovine blood. Human blood was collected in BD Vacutainer^®^ Heparin Tubes (Becton Dickinson, New Jersey, USA) from volunteers who provided informed consent. The experiments involving human blood were approved by the Human Ethics Committee of Rondônia University (CAAE 48038821.7.0000.5300).

### 2.3. Anesthesia Procedures and Blood Collection

**Figure 1:**
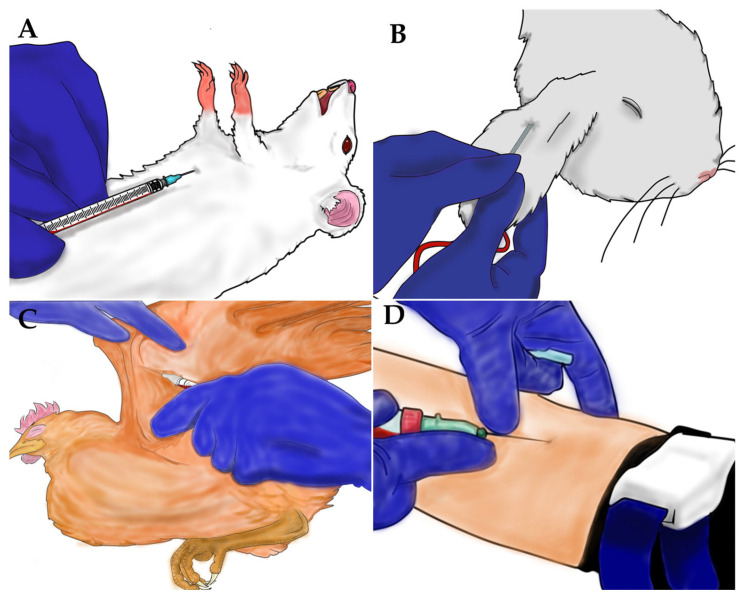
Blood collection of samples from the different sources. (**A**) blood collection via cardiac puncture in mice; (**B**) blood collection via venous puncture in rabbits, in (**C**) chickens and in (**D**) humans.

In the experiments where animals were anesthetized, a dose of ketamine (Syntec, Barueri, São Paulo, Brazil) and xylazine (Syntec, Barueri, São Paulo, Brazil) was administered intramuscularly (rabbit: 35 mg/kg ketamine and 5 mg/kg xylazine; chicken: 75 mg/kg ketamine and 6 mg/kg xylazine). For blood collection in the blood source experiments, the same doses of ketamine and xylazine were applied, with the dosage for mice being adjusted to 100 mg/kg ketamine and 10 mg/kg xylazine. Blood samples from mice were collected via cardiac puncture (Figure 1A), while blood from rabbits (Figure 1B), chickens (Figure 1C), and humans (Figure 1D) were collected through venous puncture. Anesthetizing animals for blood collection is essential to minimize discomfort and stress, ensuring adherence to good animal experimentation practices. It also safeguards the technician by avoiding the risk of accidents during the procedure. In contrast, blood collection in humans is quick and relatively painless. Volunteers are provided with informed consent and have the option to refuse the procedure if they experience discomfort or fear.

Initially, the blood samples were collected using tubes with 3.2% sodium citrate as an anticoagulant (VACUETTE^®^ Coagulation Sodium Citrate/CTAD Tubes, Greiner Bio-One, Rainbach im Mühlkreis, Austria). However, during the first two repetitions of the experiment, a high mortality of mosquitoes was observed in the experimental groups, which made it impossible to continue with the experiment. Subsequently, the blood samples were collected in tubes with heparin as the anticoagulant to prevent coagulation and were kept at room temperature in a homogenizer until the experiment began.

### 2.4. Anopheles darlingi Rearing

Only female mosquitoes were used in this study and were obtained from the *An. darlingi* colony at the Malaria Vectors Production and Infection Platform (PIVEM), maintained at Fiocruz Rondônia. The colony was maintained with rabbit blood provided by direct feeding under anesthesia once a week to produce new generations (CEUA number 2019/10). Larvae were reared on ground TetraMin^®^ Marine fish food (Tetra GmbH, Melle, Germany), while adult mosquitoes were fed a 15% honey solution source from a local producer (PROVE, Vilhena/Rondônia/Brazil) [11].

Adult mosquitoes were kept in cages (35 cm × 35 cm × 35 cm) under standard insectary conditions (26 ± 1 °C, 70 ± 10% relative humidity, with a 12:12 h light–dark photoperiod). Three-to-five-day-old females were used for experiments, and 8 h prior to blood feeding, flasks containing the 15% honey solution were removed from the cages, as fasting stimulates the mosquitoes’ search for a blood meal. Females from generations F18 to F20 were used in direct blood feeding experiments; F36 to F46 for anesthesia versus physical restraint experiments; and F48 to F50 for the indirect blood-feeding experiments.

### 2.5. Blood Feeding

**Figure 2:**
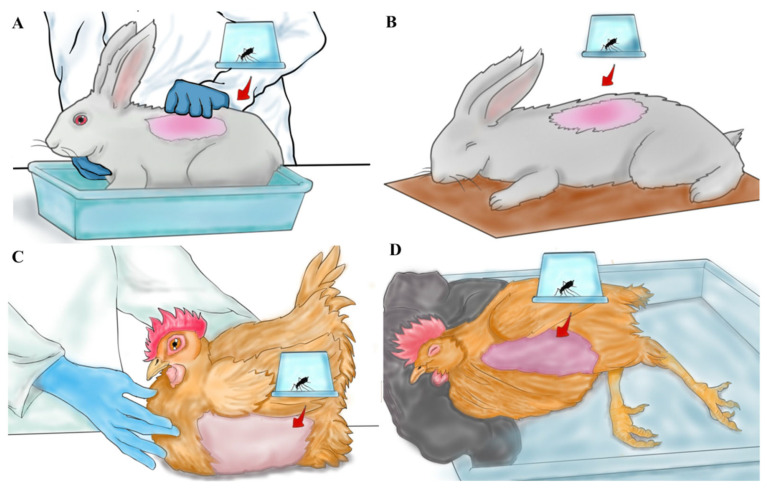
Blood feeding on rabbits and chickens. Mosquito cages were positioned over the shaved area of the animals to allow for blood feeding. (**A**) A non-anesthetized rabbit was physically restrained during the procedure. (**B**) An anesthetized rabbit was placed on wood paper, enduring a proper position to avoid the obstruction of the animal’s airways. (**C**) For the blood meal on the chicken, feathers were removed from the chest area before positing the mosquito cages. (**D**) A piece of felt fabric was used to maintain the head of the anesthetized chicken at a medium-high position, which is essential for preserving the normal breathing and blood circulation of the animal.

To assess the effects of the anesthesia, eight cages containing 100 female mosquitoes each were prepared for the blood feeding of non-anesthetized (Figure 2A,B) and anesthetized (Figure 2C,D) rabbits and chickens, with two cages per experimental group. Each cage was considered a biological unit of replication. A piece of felt fabric was used to maintain the head of the anesthetized chicken in a slightly elevated position, which is crucial for ensuring normal breathing and blood circulation (Figure 2D). All experimental groups underwent blood feeding simultaneously for 15 min. Afterwards, only fully fed mosquitoes were maintained for the experiment, while partially fed or unfed mosquitoes were discarded.

To assess the effect of blood source on the reproductive potential of *An. darlingi* through direct feeding, six cages containing 100 females were prepared for blood feeding on humans, rabbits, and chickens (two cages per blood source). Each cage was considered a biological unit of replication. Rabbits and chickens were physically restrained as mentioned before (Figure 2A,B), and for human feeding, a volunteer exposed their arm to the mosquitoes. Blood feeding lasted for 15 min.

**Figure 3:**
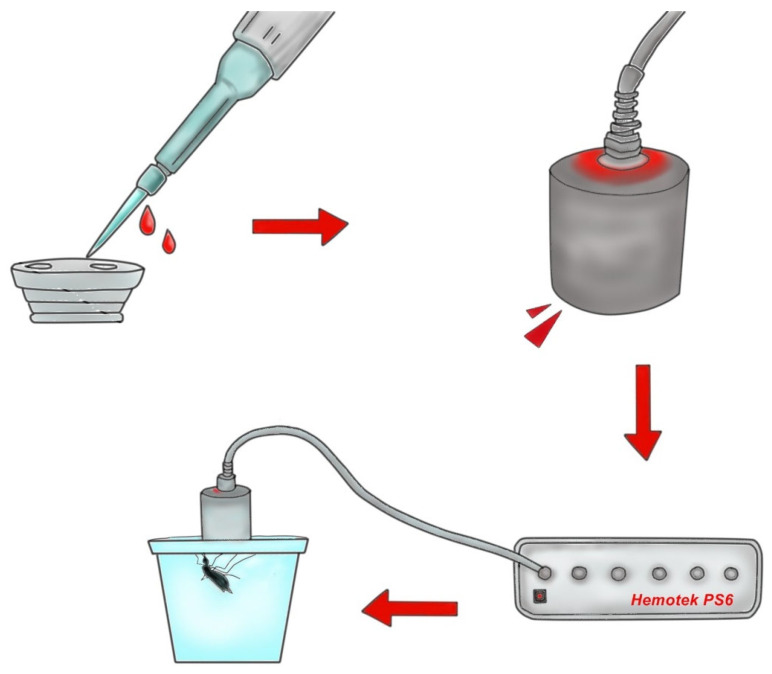
Procedure for indirect blood feeding. Blood was placed in the feeding disc of the Hemotek system, which was sealed at the bottom with Parafilm-M. The disc was attached to the feeding system to maintain the temperature at 37 °C.

For indirect blood feeding, five cages containing 100 female mosquitoes were prepared (one cage per blood source: human, mouse, bovine, rabbit, and chicken). Each cage was considered an experimental unit. Blood was collected the same day as the blood feeding experiment. Artificial blood feeding was performed using a Hemotek membrane blood feeder (PS-6 System, Discovery Workshops, Accrington, UK). A piece of Parafilm-M (Bemis Company, Inc., Neenah, Wisconsin, EUA) was stretched across the Hemotek feeder to simulate skin for mosquito feeding (Figure 3). Two milliliters of blood from each source were used, and the mosquitoes were allowed to feed for 30 min. Only fully fed mosquitoes were included in the subsequent experiments.

### 2.6. Biological Parameters Analyzed

The number of fully blood-fed mosquitoes was counted, and the blood-feeding rate was calculated by dividing the number of blood-fed mosquitoes by the total number of mosquitoes tested, then multiplying by 100%.

The survival rates of engorged females after feeding on different blood sources and under various experimental conditions were recorded daily until day four, when the females laid their eggs. On day four post-blood meal, a black plastic cup containing 50 mL of distilled water, with its walls lined with moist filter paper, was placed inside the cages to collect the eggs. On day seven post-blood meal, the cups were removed, and the number of eggs was counted using a Leica Lenz L4 stereo microscope (Leica Microsystems, Heerbrugg, Switzerland) to determine fecundity.

All eggs were transferred to white plastic rearing trays (meas. 29.1 cm × 23.0 cm × 5.0 cm) containing 1 L of distilled water, and the number of larvae was recorded to determine the hatching rate. The larvae were counted on day five after being placed in the trays.

To assess the pupation and emergence rate for each blood source and experimental condition, two plastic rearing trays with 200 larvae per tray were used, and development was monitored until the adult emergence. The pupation and adult emergence were recorded. The larvae were reared to the pupal stage under the same conditions used for maintaining the *An. darlingi* colony [11].

### 2.7. Statistical Analysis

All the data were registered in Excel (Windows version 14.0), and statistical analyses were performed using GraphPad Prism (version 8.0). Normality of the data was checked using the Shapiro–Wilk test. For comparisons between the anesthesia against physically restrained groups, an unpaired t-test were used to analyze biological parameters. A one-way analysis of variance (ANOVA) test followed by Tukey’s test, or Kruskal–Wallis tests followed by Dunn’s multiple comparisons test, were used to compare the blood sources in the direct and indirect feeding experiments.

## 3. Results

### 3.1. The Effect of Anesthesia on the Reproductive Potential of Anopheles darlingi

The reproductive potential of An. darlingi was assessed using 1524 female mosquitoes that were fully engorged on chickens and 1257 fully engorged on rabbits, either under the anesthetized or non-anesthetized condition. A higher proportion of engorged females were observed with anesthetized rabbits, likely due the more stable behavior of anesthetized animals compared with physically restrained rabbits (Table 1). In chickens, there was a slight tendency for a higher proportion of engorged females and greater fecundity in non-anesthetized chickens (Table 1). However, none of the biological parameters evaluated for either animals showed significant differences between anesthetized or non-anesthetized groups (Table 1).

### 3.2. Blood Source Effect by Direct and Indirect Blood Feeding

To evaluate the effect of blood source by direct feeding, 2039 fully engorged female mosquitoes that fed on human, rabbit, and chicken blood were monitored. No significant differences were observed in the biological parameters assessed for each blood source provided by direct feeding (Table 2).

For indirect blood feeding, 910 females completely engorged on blood source were kept for the experiments. No significant differences were observed among the biological parameters assessed from each blood source provided through indirect blood feeding (Table 3).

## 4. Discussion

Establishing a colony of *An. darlingi* is essential to support malaria research in the Brazilian Amazon, particularly in addressing the challenges posed by vivax malaria. To achieve this, it is crucial to ensure the reproductive potential of mosquitoes under laboratory conditions, enabling the production of large numbers of individuals for colony maintenance and research proposes. Because female mosquitoes require blood meals to develop their offspring, this study aimed to identify an appropriate blood source for maintaining *An. darlingi* in the laboratory. Experiments were performed to assess the effects of animal anesthesia and blood source, provided through both direct and indirect blood feeding, on the reproductive potential of *An. darlingi* females. Overall, the results indicated that neither anesthesia nor blood sources were not important factors for maintaining an *An. darlingi* colony.

Typically, blood-feeding activities for mosquito colony maintenance are performed on live animals [36]. The *An. darlingi* colony had been fed on chickens or rabbits that were physically restrained [11]. The results indicated that chicken and rabbit blood did not show significant differences compared with human blood for *An. darlingi* production. However, animals subjected to feeding in *An. darlingi* cages through physical restraint exhibited considerable behavioral instability after a period, likely due to the discomfort and stress associated with blood feeding. To minimize animal discomfort, anesthesia was administered before blood feeding, and its effects on the reproductive potential of *An. darlingi* were assessed. While anesthetics did not demonstrate clear negative effects on *An. darlingi* reproduction, some adverse effects were observed in the anesthetized animals. Chicken exhibited excessive and unusual salivation, while rabbits developed pasty feces, even with the rotation of animals between experiments, as recommend by CEUA guidelines. It is well documented that anesthetized animals often experience significant drops in core body temperature. Although the temperature of the anesthetized animals was not recorded in our study, no noticeable effect on *An. darlingi* feeding behavior was observed. This contrasts with the findings of a previous study, which reported significant impact on *An. stephensi* feeding behavior when using anesthetized guinea pigs [37].

An alternative method for offering blood meal to mosquitoes is through indirect feeding, using blood collected from live animals [38,39]. However, this method also relies on the availability of live animals, requiring a bioterium and specialized personnel for blood collection [36]. The results presented here indicate that using cattle blood from slaughterhouses is a good alternative for maintaining *An. darlingi* in the laboratory, as no significant differences were observed among the five blood sources tested for indirect blood feeding.

A previous study demonstrated that bovine blood was more efficient and practical than chicken and human blood in experiments with *Aedes aegypti* [44]. They suggested that bovine blood should be used as an appropriate blood meal source for rearing *Aedes* mosquitoes compared with the other blood sources tested. Some studies have shown that mosquitoes fed on chickens tend to produce more eggs compared with those fed on mammalian blood, likely dues to the higher nutritional content of nucleated erythrocytes, which are more beneficial for egg formation [44,45]. However, this effect was not observed in the present experiments.

Although no significant differences were observed among the tested blood sources, the practicality of obtaining large quantities of bovine blood (200 mL) and its similar performance to other blood sources in supporting the reproductive potential of *An. darlingi* led to the selection of bovine blood as the standard for maintaining the *An. darlingi* colony. It is important to note that bovine blood poses a lower risk of contamination for both handlers and colony maintenance. The cattle reared in Rondônia are vaccinated, monitored, and inspected by municipal and state health agencies to ensure the production of healthy, suitable meat for human consumption.

In nature, *An. darlingi* is primarily anthropophilic, but several studies have provided strong evidence of its opportunistic feeding behavior [40,42,46]. A preference for bovine blood have been observed in *An. darlingi* populations across the Brazilian Amazon. For example, in Belém/Para, Brazil, a high preference for human blood (49%) was recorded, followed by bovine blood (30%) [43]. Similarly, in Ariquemes/Rondonia, Brazil, a preference for human blood was followed by bovine blood. In areas of Amapá, Brazil, where bovine blood source was abundant, a lower proportion of human blood preference was observed in *An. darlingi* [47]. More recently, this trend was also documented in localities in Acre and southern Amazonas state [42].

In the Peruvian Amazon, *An. darlingi* populations demonstrated a strong preference for human hosts followed by Galliformes hosts (chickens and turkeys) [40]. Zoophilic preferences were also observed in Colombian regions, where pigs, dogs, and Galliformes were preferred over humans [48]. Interestingly, Nagaki et al. [42] noted that despite the abundance of chickens in peridomestic environments in the Brazilian Amazon, *An. darlingi* showed less preference for them.

Several studies have highlighted a population structure in *An. darlingi* across the Amazon, including clusters in the Brazilian and Peruvian Amazon, which may contribute to genetic differentiation [49,50]. These genetic differences between *An. darlingi* populations could influence ecological and behavioral traits, potentially impacting malaria control strategies [51]. The high plasticity in host source preference observed in *An. darlingi* populations may reflect this genetic population structure [40,42].

The present study was not designed to assess host preference in *An. darlingi*, and therefore, the results do not definitive conclusions about host association in the studied population. However, they provide insights into blood sources’ effects on entomological parameters, such fecundity and survival during the gonotrophic cycle, in this laboratory-colonized population. Future attempts to colonize *An. darlingi* from different populations should consider the behavior patterns and food preferences of the parental population to ensure optimal colony adaptation. Further studies could explore the impacts of blood sources and host associations on mosquito survival to gain a better understanding of malaria transmission risk in endemic areas.

## 5. Conclusions

Maintaining a highly productive mosquito colony requires ensuring the reproductive potential of the mosquitoes. The availability of the blood source is also crucial, especially when aiming to eliminate direct blood feeding on live animals and maintaining them in the institution. The practicality of obtaining blood samples must be considered, and in this regard, bovine blood proves to be an effective alternative for maintaining an *An. darlingi* colony. Additionally, *An. darlingi* appears to be relatively generalist in its feeding behavior under laboratory-rearing conditions. It may be beneficial to assess the blood source preferences of wild populations before adapting the blood source for colony maintenance in the laboratory.

## Figures and Tables

**Table 1:** Biological parameters assessed, comparing anesthesia vs. physical restraint in rabbits and chickens.

Parameters	Initial n	Biological Replicates	Rabbit		Chicken	
			Anesthetized (±SEM)	Non-Anesthetized (±SEM)	Anesthetized (±SEM)	Non-Anesthetized (±SEM)
Feeding rate (%)	100 females	2	80.8 (2.8)	76.4 (2.5)	72.0 (8.0)	80.3 (7.8)
Survival rate (%)		2	92.4 (2.9)	94.8 (2.0)	87.5 (4.8)	92.0 (2.8)
Fecundity (mean of eggs/female)		2	36.3 (6.2)	35.7 (6)	15.4 (5.7)	19.6 (5.9)
Hatching rate (%)		2	70.8 (7.0)	79.7 (4.2)	64.4 (5.1)	64.1 (5.0)
Pupation rate (%)	200 larvae	2	75.4 (8.2)	80.6 (6.9)	54.8 (4.5)	66.3 (10.4)
Adult emergence rate (%)		2	93.6 (1.8)	91.6 (1.9)	-	-

**Table 2:** Biological parameters assessed by different blood sources in the direct blood-feeding experiments.

Parameters	Initial n	Biological Replicates	Blood Type			*p*-Value (ANOVA)
			Human (±SEM)	Chicken (±SEM)	Rabbit (±SEM)	
Feeding rate (%)	100 females	2	92.6 (4.0)	88.8 (6.2)	77.2 (6.8)	0.200
Survival rate (%)	-	2	94.1 (1.8)	86.8 (5.7)	91.2 (2.3)	0.411
Fecundity (mean of eggs/female)	-	2	11.5 (3.0)	12.3 (2.1)	12.9 (3.3)	0.943
Hatching rate (%)	-	2	54.4 (9.0)	49.8 (10.5)	50.4 (12.2)	0.947
Pupation rate (%)	200 larvae	2	78.7 (2.9)	72.5 (2.5)	83.5 (4.9)	0.150
Adult emergence rate (%)	-	2	97.4 (1.4)	96.5 (1.5)	97.1 (1.5)	0.908

**Table 3:** Biological parameters of blood-feeding sources under indirect blood feeding.

Parameters	Initial n	Blood Type					*p*-Value (ANOVA)
		Mice (±SEM)	Rabbit (±SEM)	Chicken (±SEM)	Bovine (±SEM)	Human (±SEM)	
Feeding rate (%)	100 females	72.4 (17.0)	75.0 (3.5)	75.4 (0.4)	80.5 (6.6)	74.1 (7.4)	0.920 ^K^
Survival rate (%)	-	90.8 (3.4)	98.2 (0.3)	97.9 (0.4)	83.7 (13.4)	96.5 (1.0)	0.448
Fecundity (mean of eggs/female)	-	100.2 (2.5)	74.6 (9.6)	67.0 (13.7)	88.3 (18.4)	73.9 (4.8)	0.270
Hatching rate (%)	-	76.4 (10.2)	78.2 (4.7)	74.1 (5.1)	64.1 (3.6)	73.9 (4.8)	0.557
Pupation rate (%)	400 larvae	88.1 (3.7)	85.6 (6.0)	91.5 (3.6)	78.9 (10.0)	88.4 (5.3)	0.675
Adult emergence rate (%)	-	98.3 (0.10)	98.2 (0.6)	98.6 (0.2)	98.6 (0.5)	99.0 (0.3)	0.667

^K^ It shows the *p*-value from the Kruskal–Wallis test.

## Data Availability

Data are contained within the article or Supplementary Materials.

## Supplementary Materials

The following supporting information can be downloaded at: https://www.mdpi.com/article/10.3390/insects16030281/s1, Table S1: Raw data from the experiments performed.

## References

[1] Rougeron V., Boundenga L., Arnathau C., Durand P., Renaud F., Prugnolle F. (2022). A population genetic perspective on the origin, spread and adaptation of the human malaria agents *Plasmodium falciparum* and *Plasmodium vivax*. FEMS Microbiol. Rev..

[2] World Health Organization Malaria. 11 December 2024. https://www.who.int/news-room/fact-sheets/detail/malaria.

[3] Angrisano F., Robinson L.J. (2022). *Plasmodium vivax*—How hidden reservoirs hinder global malaria elimination. Parasitol. Int..

[4] Gonçalves P.R., Gomes F.L.R., Ribeiro C.T.D. (2022). Malaria Related Neurocognitive Deficits and Behavioral Alterations. Front. Cell. Infect. Microbiol..

[5] Vitor-Silva S., Reyes-Lecca R.C., Pinheiro T.R., Lacerda M.V. (2009). Malaria is associated with poor school performance in an endemic area of the Brazilian Amazon. Malar. J..

[6] Tapajós R., Castro D., Melo G., Balogun S., James M., Pessoa R. (2019). Malaria impact on cognitive function of children in a peri-urban community in the Brazilian Amazon. Malar. J..

[7] Devine A., Pasaribu A.P., Teferi T., Pham H.T., Awab G.R., Contantia F., Nguyen T.N., Ngo V.T., Tran T.H., Hailu A. (2019). Provider and household costs of *Plasmodium vivax* malaria episodes: A multicountry comparative analysis of primary trial data. Bull. World Health Organ..

[8] Gunalan K., Rowley E.H., Miller L.H. (2020). A Way Forward for Culturing *Plasmodium vivax*. Trends Parasitol..

[9] Moreno M., Tong C., Guzmán M., Chuquiyauri R., Llanos-Cuentas A., Rodriguez H., Gamboa D., Meister S., Winzeler E.A., Maguina P. (2014). Infection of laboratory-colonized *Anopheles darlingi* mosquitoes by *Plasmodium vivax*. Am. J. Trop. Med. Hyg..

[10] Villarreal-Trevino C., Vasquez G.M., Lopez-Sifuentes V.M., Escobedo-Vargas K., Huayanay-Repetto A., Linton Y.M., Flores-Mendoza C., Lescano A.G., Stell F.M. (2015). Establishment of a free-mating, long-standing and highly productive laboratory colony of *Anopheles darling* from the Peruvian Amazon. Malar. J..

[11] Araujo M.d.S., Andrade A.O., Santos N.A.C.d., Pereira D.B., Costa G.d.S., Paulo P.F.M.d., Rios C.T., Moreno M., Pereira-da-Silva L.H., Medeiros J.F. (2019). Brazil’s first free-mating laboratory colony of *Nyssorhynchus darlingi*. Rev. Soc. Bras. Med. Trop..

[12] Puchot N., Lecoq M.-T., Carinci R., Duchemin J.B., Gendrin M., Bourgouin C. (2022). Establishment of a colony of *Anopheles darlingi* from French Guiana for vector competence studies on malaria transmission. Front. Trop..

[13] Póvoa M.M., de Souza R.T.L., Lacerda R.N.d.L., Rosa E.S., Galiza D., de Souza J.R., A Wirtz R., Schlichting C.D., E Conn J. (2006). The importance of *Anopheles albitarsis* E and *An. darlingi* in human malaria transmission in Boa Vista, state of Roraima, Brazil. Mem. Do Inst. Oswaldo Cruz.

[14] Andrade A.O., dos Santos N.A.C., Castro R.B., de Araujo I.S., Bastos A.d.S., Magi F.N., Rodrigues M.M.d.S., Pereira D.B., Medeiros J.F., Araújo M.d.S. (2021). Description of malaria vectors (Diptera: Culicidae) in two agricultural settlements in the Western Brazilian Amazon. Rev. Inst. Med. Trop. São Paulo.

[15] Vittor A.Y., Pan W., Gilman R.H., Tielsch J., Glass G., Shields T., Sánchez-Lozano W., Pinedo V.V., Salas-Cobos E., Flores S. (2009). Linking deforestation to malaria in the Amazon: Characterization of the breeding habitat of the principal malaria vector, *Anopheles darlingi*. Am. J. Trop. Med. Hyg..

[16] Bastos A.S., dos Santos N.A.C., Andrade A.O., Pontual J.D.C., Araújo J.E., Medeiros J.F., Araujo M.S. (2024). Evaluation of insemination, blood feeding, and *Plasmodium vivax* infection effects on locomotor activity patterns of the malaria vector *Anopheles darlingi* (Diptera: Culicidae). Parasitol. Res..

[17] Araujo M.S., Guo F., Rosbash M. (2020). Video Recording Can Conveniently Assay Mosquito Locomotor Activity. Sci. Rep..

[18] Santos N.A.C., Martins M.M., Andrade A.O., Bastos A.S., Pontual J.D.C., Araújo J.E., Rocha M.L., Medeiros J.F., Araujo M.S. (2024). Effects of Carbohydrate Intake on *Anopheles darlingi* and *Anopheles deaneorum* Fitness Under Lab-Reared Conditions. Insects.

[19] Acford-Palmer H., Andrade A.O., Phelan J.E., Santana R.A., Lopes S.C.P., Medeiros J.F., Clark T.G., Araujo M.S., Campino S. (2025). Application of a targeted amplicon sequencing panel to screen for insecticide resistance mutations in *Anopheles darlingi* populations from Brazil. Sci. Rep..

[20] Penna-Coutinho J., Araujo M.S., Aguiar A.C.C., Sá P.M., Rios C.T., Medeiros J.F., Pereira D.B., Boechat N., Krettli A.U. (2021). MEFAS, a hybrid of artesunate-mefloquine active against asexual stages of *Plasmodium vivax* in field isolates, inhibits malaria transmission. Int. J. Parasitol. Drugs Drug Resist..

[21] Calit J., Araújo J.E., Deng B., Miura K., Gaitán X.A., Araujo M.S., Medeiros J.F., Long C.A., Simeonov A., Eastman R.T. (2023). Novel Transmission-Blocking Antimalarials Identified by High-Throughput Screening of *Plasmodium berghei* Ookluc. Antimicrob. Agents Chemother..

[22] Bansal G.P., Araujo M.S., Cao Y., Shaffer E., Araujo J.E., Medeiros J.F., Hayashi C., Vinetz J., Kumar N. (2024). Transmission-reducing and -enhancing monoclonal antibodies against *Plasmodium vivax* gamete surface protein Pvs48/45. Infect. Immun..

[23] Khan J., Gholizadeh S., Zhang D., Wang G., Guo Y., Zheng X., Wu Z., Wu Y. (2022). Identification of a biological form in the *Anopheles stephensi* laboratory colony using the odorant-binding protein 1 intron I sequence. PLoS ONE.

[24] Santos N.A.C., Andrade A.O., Santos T.C., Martinez L.N., Ferreira A.S., Bastos A.S., Martins M.M., Pontual J.D.C., Teles C.B.G., Medeiros J.F. (2022). Evaluation of sustainable susceptibility to *Plasmodium vivax* infection among colonized *Anopheles darlingi* and *Anopheles deaneorum*. Malar. J..

[25] Santos N.A.C., Magi F.N., Andrade A.O., Bastos A.S., Pereira S.S., Medeiros J.F., Araujo M.S. (2022). Assessment of antibiotic treatment on *Anopheles darlingi* survival and susceptibility to *Plasmodium vivax*. Front. Microbiol..

[26] Benedict M.Q., Knols B.G., Bossin H.C., I Howell P., Mialhe E., Caceres C., Robinson A.S. (2009). Colonization and mass rearing: Learning from others. Malar. J..

[27] Williams J., Flood L., Praulins G., Ingham V.A., Morgan J., Less R.S., Hanson H. (2009). Characterization of *Anopheles* strains used for laboratory screening of new vector control products. Parasites Vectors.

[28] Facchinelli L., Valerio L., Lees R.S., Oliva C.F., Persampieri T., Collins C.M., Crisanti A., Spaccapelo R., Benedict M.Q. (2015). Stimulating *Anopheles gambiae* swarms in the laboratory: Application for behavioral and fitness studies. Malar. J..

[29] Villarreal C., Arredondo-Jiménez J.I., Rodriguez M.H., Ulloa A. (1998). Colonization of *Anopheles pseudopunctipennis* from Mexico. J. Am. Mosq. Control Assoc..

[30] Baker R.H. (1964). Mating problems as related to the establishment and maintenance of laboratory colonies of mosquitos. Bul. World Health Org..

[31] Phasomkusolsil S., Tawong J., Monkanna N., Pantuwatana K., Damdangdee N., Khongtak W., Kertmanee Y., Evans B.P., Schuster A.L. (2013). Maintenance of mosquito vectors: Effects of blood source on feeding, survival, fecundity, and egg hatching rates. J. Vector Ecol..

[32] Dias L.d.S., da Bauzer L.G.S.R., Lima J.B.P. (2018). Artificial blood feeding for Culicidae colony maintenance in laboratories: Does the blood source condition matter?. Rev. Inst. Med. Trop. Sao Paulo.

[33] Harrington L.C., Edman J.D., Scott T.W. (2001). Why do female *Aedes aegypti* (Diptera: Culicidae) feed preferentially and frequently on human blood?. J. Med. Entomol..

[34] Turell M.J. (1988). Reduced Rift Valley fever virus infection rates in mosquitoes associated with pledget feedings. Am. J. Trop. Med. Hyg..

[35] Thomas J.A., Bailey D.L., Dame D.A. (1985). Maintenance of *Anopheles albimanus* on frozen blood. J. Am. Mosq. Control. Assoc..

[36] Benedict M.Q. (2015). Methods in Anopheles Research.

[37] Buchta J.N., Zarndt B.S., Garver L.S., Rowland T., Shi M., Davidson S.A., Rowton E.D. (2015). Blood-Feeding Behaviors of *Anopheles stephensi* but not *Phlebotomus papatasi* are influenced by Actively Warming Guinea Pigs (*Cavia porcellus*) Under General Anesthesia. J. Am. Mosq. Control. Assoc..

[38] Luo Y.P. (2014). A novel multiple membrane blood-feeding system for investigating and maintaining *Aedes aegypti* and *Aedes albopictus* mosquitoes. J. Vector Ecol..

[39] Gunathilaka N., Ranathunge T., Udayanga L., Abeyewickreme W. (2017). Efficacy of Blood Sources and Artificial Blood Feeding Methods in Rearing of *Aedes aegypti* (Diptera: Culicidae) for Sterile Insect Technique and Incompatible Insect Technique Approaches in Sri Lanka. Biomed Res. Int..

[40] Moreno M., Saavedra M.P., Bickersmith S.A., Prussing C., Michalski A. (2017). Intensive trapping of blood-fed *Anopheles darlingi* in Amazonian Peru reveals unexpectedly high proportions of avian blood-meals. PLoS Negl. Trop Dis..

[41] Oliveira-Ferreira J., Lourenço-de-Oliveira R., Deane L.M., Daniel-Ribeiro C.T. (1992). Feeding preference of *Anopheles darlingi* in malaria endemic areas of Rondônia state, northwestern Brazil. Mem. Inst. Oswaldo Cruz.

[42] Nagaki S.S., Chaves L.S.M., López R.V.M., Bergo E.S., Laporta G.Z., Conn J.E., Sallum M.A.M. (2021). Host feeding patterns of *Nyssorhynchus darlingi* (Diptera: Culicidae) in the Brazilian Amazon. Acta Trop..

[43] Deane L., Vernin C.S., Damasceno R.G. (1949). Avaliação das preferências alimentares das fêmeas de *Anopheles darlingi* e *Anopheles aquasalis* em Belém, Pará, por meio de Provas de precipitina. Rev. Serv. Esp. Saúde Públ..

[44] Bennett G.F. (1970). The influence of the blood meal type on the fecundity of *Aedes aegypti* L. (Stegomyia). (Diptera: Culicidae). Can. J. Zool..

[45] Harrison R.E., Brown M.R., Strand M.R. (2021). Whole blood and blood components from vertebrates differentially affect egg formation in three species of anautogenous mosquitoes. Parasites Vectors.

[46] Altamiranda-Saavedra M., Conn J.E., Correa M.M. (2017). Genetic structure and phenotypic variation of *Anopheles darlingi* in northwest Colombia. Infect. Genet. Evol..

[47] Zimmerman R.H., Galardo A.K.R., Lounibos L.P., Arruda M., Wirtz R. (2006). Bloodmeal Hosts of *Anopheles* Species (Diptera: Culicidae) in a Malaria-Endemic Area of the Brazilian Amazon. J. Med. Entomol..

[48] Piedrahita S., Álvarez N., Naranjo-Díaz N., Bickersmith S., Conn J.E., Correa M.M. (2022). *Anopheles* blood meal sources and entomological indicators related to *Plasmodium* transmission in malaria endemic areas of Colombia. Acta Trop..

[49] Mirabello L., Vineis J.H., Yanoviak S.P., Scarpassa V.M., Póvoa M.M., Padilla N., Achee N.L., E Conn J. (2008). Microsatellite data suggest significant population structure and differentiation within the malaria vector *Anopheles darlingi* in Central and South America. BMC Ecol..

[50] Emerson K.J., Conn J.E., Bergo E.S., Randel M.A., Sallum M.A.M. (2015). Brazilian *Anopheles darlingi* Root (Diptera: Culicidae) Clusters by Major Biogeographical Region. PLoS ONE..

[51] Conn J.E., Ribolla P.E., Adelman Z.N. (2016). Ecology of *Anopheles darlingi*, the Primary Malaria Vector in the Americas and Current Nongenetic Methods of Vector Control. Genetic Control of Malaria and Dengu.

